# Role of Fruit Epicuticular Waxes in Preventing *Bactrocera oleae* (Diptera: Tephritidae) Attachment in Different Cultivars of *Olea europaea*

**DOI:** 10.3390/insects11030189

**Published:** 2020-03-17

**Authors:** Manuela Rebora, Gianandrea Salerno, Silvana Piersanti, Elena Gorb, Stanislav Gorb

**Affiliations:** 1 Department of Chemistry, Biology and Biotechnology, University of Perugia, Via Elce di Sotto 8, 06121 Perugia, Italy; manuela.rebora@unipg.it (M.R.); silvana.piersanti@unipg.it (S.P.); 2 Department of Agricultural, Food and Environmental Sciences, University of Perugia, Borgo XX Giugno, 06121 Perugia, Italy; 3 Department of Functional Morphology and Biomechanics, Zoological Institute, Kiel University, Am Botanischen Garten 9, 24098 Kiel, Germany; egorb@zoologie.uni-kiel.de (E.G.); sgorb@zoologie.uni-kiel.de (S.G.)

**Keywords:** friction, adhesion, olive fruit fly, pulvilli, claws, wax crystals, biomechanics

## Abstract

The olive fruit fly *Bactrocera oleae* (Diptera: Tephritidae) is the major pest of cultivated olives (*Olea europaea* L.), and a serious threat in all of the Mediterranean Region. In the present investigation, we demonstrated with traction force experiments that *B. oleae* female adhesion is reduced by epicuticular waxes (EWs) fruit surface, and that the olive fruit fly shows a different ability to attach to the ripe olive surface of different cultivars of *O. europaea* (Arbequina, Carolea, Dolce Agogia, Frantoio, Kalamata, Leccino, Manzanilla, Picholine, Nostrale di Rigali, Pendolino and San Felice) in terms of friction force and adhesion, in relation with different mean values of olive surface wettability. Cryo-scanning morphological investigation revealed that the EW present on the olive surface of the different analyzed cultivars are represented by irregular platelets varying in the orientation, thus contributing to affect the surface microroughness and wettability in the different cultivars, and consequently the olive fruit fly attachment. Further investigations to elucidate the role of EW in olive varietal resistance to the olive fruit fly in relation to the olive developmental stage and environmental conditions could be relevant to develop control methods alternative to the use of harmful pesticides.

## 1. Introduction

The interaction between plants and their environment is mediated by a series of complex chemical and physical factors, among which epicuticular waxes (EWs), covering the surface of most plant organs, have a fundamental functional role [1,2]. EWs are a complex mixture of cyclic (triterpenoids) or long chain aliphatic substances, such as primary and secondary alcohols, primary aldehydes, fatty acids and alkenes [1,3], and constitute two-dimensional films/layers or three-dimensional micro- or nanoscale projections covering the plant cuticle [4]. EWs represent the primary barrier against biotic and abiotic stress, exhibiting a multitude of functions, such as being a barrier against water loss [5], offering protection against incident radiation by favoring light reflection [6], protection from surface contamination by dust particles [7,8] or from pathogenic microorganisms [9].

Because of the long coevolution between plants and insects, EWs have an important role also in mediating the insect–plant interaction by protecting the plant from herbivores, or helping it in the capture of pollinators, and preventing the escape of prey insects from carnivorous plants (see review in [10]). In this regard, wax projections can decrease the ability of insects to attach to the plant cuticle surface (see review in [11,12]).

The fly family Tephritidae contains nearly 4500 known species, including some of the world’s most significant agricultural insect pests, among which the olive fruit fly *Bactrocera oleae* (Rossi) (Diptera: Tephritidae). This fly is the major pest of commercial olives worldwide, and represents a major pest in the Mediterranean basin (see reviews in [13,14]). The larvae are monophagous on olive fruit in the genus *Olea*, including *Olea europaea* L. (cultivated and wild). On both cultivated and wild olives, females of *B. oleae* lay their eggs in ripening and ripe fruit, right beneath the olive epicarp, providing direct access to food for larvae just after their egg emergence. Larvae feed upon the olive pulp, thus causing losses of up to 80% of the oil value [15]. Although *B. oleae* is a key pest in the olive crop, some *O. europaea* cultivars are less susceptible to *B. oleae* adult females [14,15,16,17]. Based upon the investigations on the susceptibility of different olive cultivars to *B. oleae* [18,19,20], it was concluded that female attraction toward the different olive cultivars is due to the interaction of several physical (e.g., fruit size, weight, volume, color) and chemical factors. Different studies reported that olives lacking the usual waxy coverage were more susceptible to olive fruit fly attack than normal olives [18,19]. In particular, Neuenschwander et al. [18] attributed this reduction of susceptibility to the thickness of the waxy coverage, able to shield the attractant chemicals in the olive cultivars. Vichi et al. [21] observed differences in the chemical composition of the EWs of nine olive varieties grown in the same geographical area, and hypothesized a possible relationship between EW composition and varietal resistance to several biotic and abiotic factors, highlighting the need of further investigations to elucidate the role of EWs in olive varietal resistance to insect pests and environmental conditions.

The EW role in reducing insect attachment to the olive surface has not been previously investigated in detail. In this study, we tested the hypothesis that the attachment of the female of *B. oleae* to the olive fruit surface can vary on the ripe fruits of different cultivars of *O. europaea*, and that this could be related to the EW features, such as wettability and nanostructure in different cultivars. In particular, we used traction force experiments to test *B. oleae* female friction and adhesion to the ripe olive surface of different cultivars of *O. europaea* (Arbequina, Carolea, Dolce Agogia, Frantoio, Kalamata, Leccino, Manzanilla, Picholine, Nostrale di Rigali, Pendolino and San Felice), and we characterized the olive surface of the different cultivars with cryo-scanning morphological investigation and contact angle measurements. 

## 2. Materials and Methods 

### 2.1. Insects

*B. oleae* adults emerged from pupae obtained from olives collected in the field around Perugia (Umbria, Italy) during October 2018. Olives were kept in the laboratory in a controlled condition chamber (14 h photophase, temperature of 25 ± 1 °C; RH of 60 ± 10%), on a net in order to collect pupae falling down. Pupae were kept inside net cages (300 mm x 300 mm x 300 mm) in a controlled condition chamber (14 h photophase, temperature of 25 ± 1 °C; RH of 60 ± 10%) until the adult emergence. Females and males after emergence were maintained in the same cages and provided with water and crystallized sucrose. Only mated females 10–15 days old were used in the experiments. 

### 2.2. Olive Fruits

Olive fruits belonging to eleven cultivars of *O. europaea* were collected in November 2018 from the germoplasm collection of the Department of Agricultural, Food and Environmental Science of the University of Perugia, located nearby Perugia town (43°04’54.58’’ N, 12°22’53.41’’ E). Healthy, ripe olive fruit samples were collected from the selected Mediterranean cultivars Arbequina, Carolea, Dolce Agogia, Frantoio, Kalamata, Leccino, Manzanilla, Picholine, Nostrale di Rigali, Pendolino and San Felice. All of the cultivars were subjected to the same cultivation practices, and kept at the same environmental conditions. Only fruits with intact EWs were used in the experiments. The investigations were carried out on ripe olives to have the possibility to compare simultaneously the attachment of *B. oleae* on different cultivars at the same level of ripeness.

### 2.3. Cryo Scanning Electron Microscopy (Cryo-SEM)

The shock-frozen samples of olive fruit surfaces of the selected cultivars and the tarsi of *B. oleae* insects (females) were studied in a scanning electron microscope (SEM) Hitachi S-4800 (Hitachi High-Technologies Corp., Tokyo, Japan) equipped with a Gatan ALTO 2500 cryo-preparation system (Gatan Inc., Abingdon, UK). For details of sample preparation and mounting for cryo-SEM, see Gorb and Gorb [22]. Whole mounts of olive fruit surface pieces and insect tarsi were sputter-coated in frozen conditions with gold-palladium (thickness 10 nm), and examined at 3 kV acceleration voltage and temperature of −120 °C at the cryo-stage within the microscope.

### 2.4. Evaluation of Pulvilli Area

To evaluate the area of pulvilli in both sexes of *B. oleae*, the adults’ tarsi were dissected from 20 anesthetized insects (10 males and 10 females), mounted on glass slides with a drop of glycerine, and observed with reflection interference contrast microscopy (RICM) using an inverted bright-field microscope ZEISS Axio Observer A1 (Carl Zeiss Microscopy GmbH, Jena, Germany). Areas of the pulvilli were measured from pad digital images taken with a Sony 3CCD video camera, and using the open source image processing program ImageJ [23]. Measurements were made individually for all 20 insects (240 pulvilli, 12 pulvilli per insect), and recorded separately for the fore-, mid- and hindlegs.

### 2.5. Characterization of Wettability of Natural (Olive Fruits) and Artificial (Hydrophilic and Hydrophobic Glass) Surfaces

The wettability of ripe, olive fruit surfaces in different cultivars and of hydrophilic and hydrophobic glass was characterized by determining the contact angles of water (aqua millipore, droplet size = 1 µl, sessile drop method) using a high-speed optical contact angle measuring instrument OCAH 200 (Dataphysics Instruments GmbH, Filderstadt, Germany). The contact angle between a liquid and a solid is the angle within the body of the liquid formed at the gas–liquid–solid interface. If the water contact angle on a surface is < 90°, the surface is considered hydrophilic, if the contact angle is ≥ 90° the surface is hydrophobic. Ten measurements (*n* = 10) were performed for each substrate. To increase confidence in the collected data for the olive fruits, wettability measurements were repeated after one year on fruits of the same cultivars grown in the same field. To confirm that the hydrophobic coating on the glass remained over repeated use/time, water contact angle measurements were repeated at the end of the force experiments. There was no significant difference between the contact angles of water on hydrophobic glass measured before and after the experiments (*t* = 1.11; *d.f.* = 13; *p* = 0.2886).

### 2.6. Force Measurements

The experiments were performed using force measuring experimental set ups for testing the insect attachment to natural surfaces (olive fruits) and artificial surfaces (hydrophilic and hydrophobic glass). Forces were measured using the load cell force sensor FORT-10 (10 g capacity; World Precision Instruments Inc., Sarasota, FL, USA) connected to a force transducer MP 100 (Biopac Systems Ltd., Goleta, CA, USA) [24]. Data were recorded using AcqKnowledge 3.7.0 software (Biopac Systems Ltd., Goleta, CA, USA). Force values were estimated using the force–time curves as the maximum of the detected forces. Only females were tested, owing to their need to firmly attach to the fruit surface during oviposition.

Prior to the force measurements, females of *B. oleae* were weighed upon a micro-balance (Mettler Toledo AG 204 Delta Range, Greifensee, Switzerland). Experimental insects were anesthetized with carbon dioxide for 60 s, and were made incapable of flying by carefully gluing their wings together with a small droplet of melted wax. For force tests, one end of about 15 cm long human hair was fixed on the insect thorax with a droplet of melted wax. Before starting experiments, insects were left to recover for 30 min. All of the experiments were performed during the daytime at 25 ± 1 °C temperature and 60 ± 10% relative humidity.

**Figure 1:**
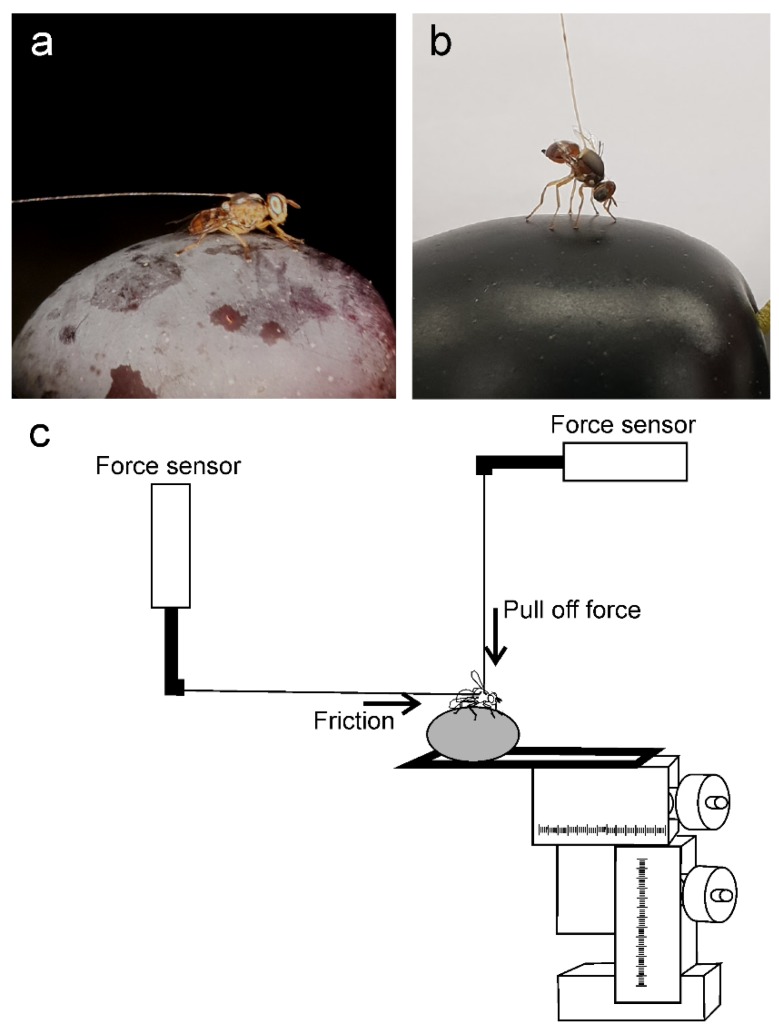
Experimental set ups for testing *Bactrocera oleae* attachment to olive fruits belonging to different cultivars. The insect is attached to the force sensor by means of a hair glued to its thorax. (**a**) Friction force measurements; (**b**) Adhesion (pull off) force measurements; (**c**) The olive fruit is attached to a microscope slide fixed to a motorized micromanipulator and a controller. To measure the friction force, the sensor (on the left side) is kept vertically and the olive is moved in a direction parallel to the olive surface and opposite to the pulling direction of an insect (horizontal arrow). To measure the pull off force, the sensor (on the right side) is kept horizontally and the olive is moved down in a direction perpendicular to the olive surface (vertical arrow).

To test insects on natural fruit surfaces, each olive fruit was firmly attached to a microscope slide, which was fixed to a motorized micromanipulator DC3314R and a controller MS314ZU (World Precision Instruments Inc., Sarasota, FL, USA). At the beginning of each experiment, the insect, attached to the force sensor by means of the hair glued to its thorax, was placed on the olive fruit surface to be tested (see Figure 1 for detail).

Two sets of experiments have been performed on natural fruit surfaces. In the first set carried out to measure the insect friction force, the sensor was kept in the vertical position, and the olive was moved in a direction parallel to the olive surface and opposite to the pulling insect until the insect detachment from the olive surface (Figure 1a,c, horizontal arrow). 

In the second set of experiments carried out to measure the insect adhesion (pull off) force, the sensor was kept in the horizontal position, and the olive was moved in a direction perpendicular to the olive surface until the insect detached from the olive surface (Figure 1b,c, vertical arrow). In both experiments, the olive was moved at a continuous speed of 200 µm s^−1^ using the motorized micromanipulator. The olive fruits were changed every three tested insects to avoid dehydration of fruits, and a possible effect of changes in EW. Each insect was tested on the olive fruit surface of each cultivar presented in a random order. Additionally, at the beginning and at the end of the sequence of olive fruits, each insect individual was tested on glass to evaluate the effect of possible reduction in insect attachment ability due to possible pulvilli contamination by eroded wax. One olive cultivar (Kalamata) was tested intact and with mechanically removed waxes (dewaxed). The waxes were mechanically removed by gently cleaning the olive fruit surface with a soft paper towel. Between the two sets of experiments, the flies were left to recover for about 30 min. In total, 15 females were tested.

In another set of force experiments for testing insect attachment to artificial surfaces (hydrophilic and hydrophobic glass), the insect attached to the force sensor by means of the hair glued to its thorax was allowed to move on the substrate to be tested in a direction perpendicular to the force sensor. The force generated by the insect walking horizontally on the test substrates (traction force) was measured. In total, 10 females were tested.

To prepare hydrophobic glass, a glass disk was rinsed and then sonicated in an ultrasound bath (Bandelin electronic, Berlin, Germany) for 5 min, first with ethanol (70%), and then with distilled water. This washing procedure was repeated several times and the remaining liquid on the substrate was blown by compressed air. Cleaned glass was subjected to air plasma treatment (Diener electronic, Ebhausen, Germany), and then vacuum pumped (BÜCHI Labortechnik, Flawil, Switzerland) in a desiccator together with a glass vial containing 200 μl of dichlorodimethylsilane (Merck Schuchardt, Hohenbrunn, Germany). The vacuum pump was disconnected from the desiccator once the silane started to boil, and the desiccator was left closed for 5 h to achieve sufficient deposition of the silane on the glass. The surface-modified glass was rinsed thoroughly with isopropanol and distilled water, and blown dry by compressed air.

### 2.7. Statistical Analysis

To compare the area of pulvilli between the sexes and among the fore-, mid- and hindlegs, two-way repeated measures analysis of variance (ANOVA), considering the insect sex and the leg type as main factors, was used. For significant factors, the Tukey’s HSD test was used as post hoc test.

The water contact angles on olive fruit surfaces were compared among the different cultivars using one-way ANOVA and the Tukey’s HSD post-hoc test for multiple comparisons between means. The water contact angles on dewaxed and intact olives of the Kalamata cultivar were compared using the *t*-test for independent samples. 

In the force experiments, the safety factors (force divided by the weight of the insect individual) obtained for the friction and pull off forces of *B. oleae* females on the fruit surfaces were analyzed with one-way repeated measures ANOVA and the Tukey’s HSD post-hoc test for multiple comparisons between means to verify differences between the tested cultivars of *O. europaea*. 

For all the performed ANOVA, F-tests were used to assess the significance of the effects and their interactions (Statistica 6.0, StatSoft Inc., Tulsa, OK, USA.).

The *t*-test for dependent samples was used to compare the safety factor values obtained on glass at the beginning and at the end of experiments with each set of olives, to compare the safety factors obtained on the fruit surface in intact fruit and fruit with mechanically removed waxes (dewaxed), and to compare the safety factors obtained on hydrophilic glass and hydrophobic glass. 

The relationship between the water contact angle and the normalized safety factor (safety factor normalized on that obtained on glass, to reduce the variability caused by individual insects), both for friction and pull off forces, was tested using linear regression analysis [25]. 

Before all the analysis, the data were subjected to Box–Cox transformations, in order to reduce data heteroscedasticity [26].

## 3. Results

### 3.1. Morphology of Bactrocera oleae Attachment Organs

**Figure 2:**
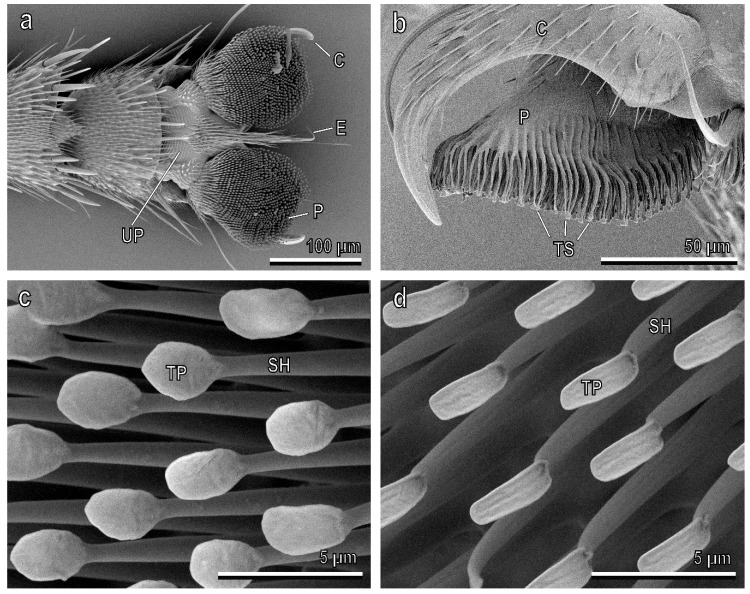
Pretarsal attachment devices of the female of *Bacrocera oleae* in the cryo-SEM. (**a**) Ventral view of hairy pulvilli (P) and curved claws (C). E, empodium, UP, unguitractor plate; (**b**) Lateral view of a pulvillus with its ventral tenent setae (TS). C, claw; (**c**) Detail of the distal tenent setae constituted of a setal shaft (SH) and a circular terminal plate (TP); (**d**) Detail of the proximal tenent setae with a narrow terminal plate (TP). SH, setal shaft.

The attachment devices of the adult *B. oleae* are located at the pretarsus, and are composed of two dorsally situated and ventrally curved claws, and two ventrally situated hairy pulvilli (Figure 2a,b). An unguitractor plate with a distal empodium consisting of a long-tapered hair is located in the basal region of the pretarsus (Figure 2a). Each pulvillus has an oval shape, and on its ventral side consists of numerous distally-oriented tenent setae (Figure 2). The dorsal side of each pulvillus is constituted of a central core, from which numerous cuticular digitations depart, giving rise to the tenent setae (Figure 2b). Each tenent seta consists of a setal shaft and a terminal plate (endplate), whose shape changes from the proximal to the distal portion of the pulvillus along its ventral side (Figure 2c,d). The distal setae have a circular terminal plate (Figure 2c), while the proximal setae have a narrower terminal plate (Figure 2d). 

The female pulvilli are wider than the male pulvilli. Moreover, in both sexes, the area of each pulvillus increases from the forelegs to the hindlegs (sex: *F* = 66.9, *d.f.* =1, 18, *p* < 0.0001; legs: *F* = 33.1, *d.f.* = 2, 36, *p* < 0.0001; sex x legs: *F* = 0.4, *d.f.* = 2, 36, *p* = 0.7036) (Table 1).

### 3.2. Surface Morphology and Wettability of Olive Fruits

**Figure 3:**
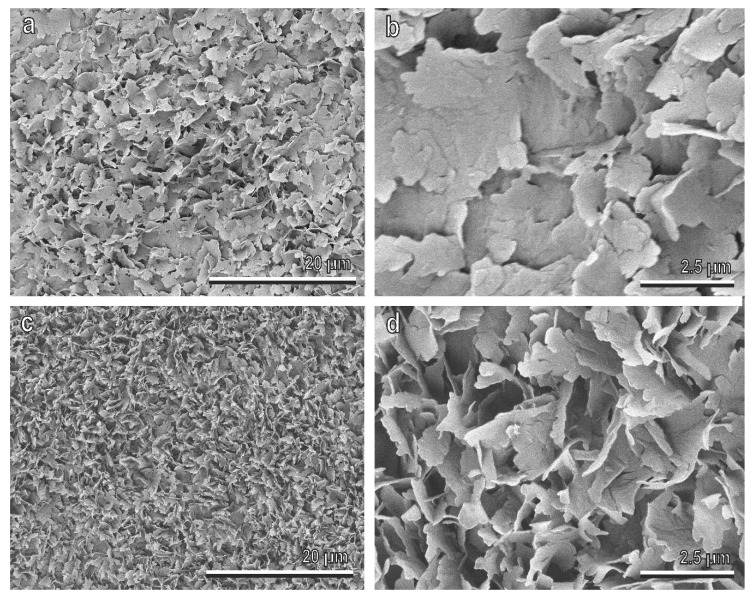
Olive (*Olea europaea*) fruit surfaces of the cultivars Manzanilla (**a**,**b**) and Picholine (**c**,**d**) in the cryo-scanning electron microscope (SEM). In (**b**) and (**d**), details of the epicuticular wax (EW) coverage composed of flat projections (platelets) with irregular sinuate margins, are shown. The platelets are attached to the fruit surface by their narrow side protruding from the surface at different angles (compare (**b**) with (**c**)).

In all studied cultivars of *O. europaea*, the ripe fruit cuticle is densely covered by EW, with a complex three-dimensional structure (Figure 3a,c). According to the classification proposed by Barthlott et al. [1], flat wax projections composing this coverage are in the form of irregular platelets, with irregular sinuate margins (Figure 3b,d). The platelets are attached to the olive fruit surface by their narrow side, and protrude from the surface at different angles. In most cases, they are oriented nearly perpendicular to the fruit surface, such as in the Picholine cultivar (Figure 3c,d), creating by this prominent fine surface roughness, but in some cultivars (e.g., Manzanilla), they have rather shallow angles with the fruit surface (Figure 3a,b) that leads to a more flattened surface profile. 

The olive fruit surface in all tested cultivars is hydrophobic, with a water contact angle statistically differing among the cultivars (*F* = 13.86, *d.f.* = 10, 95, *p* < 0.001), ranging from 102.3° in the Manzanilla cultivar to 147.6° in the Picholine cultivar (Table 2). 

### 3.3. Attachment Ability of Bactrocera oleae Females to the Olive Fruit Surface of Different Cultivars and to Hydrophilic and Hydrophobic Glass

**Figure 4:**
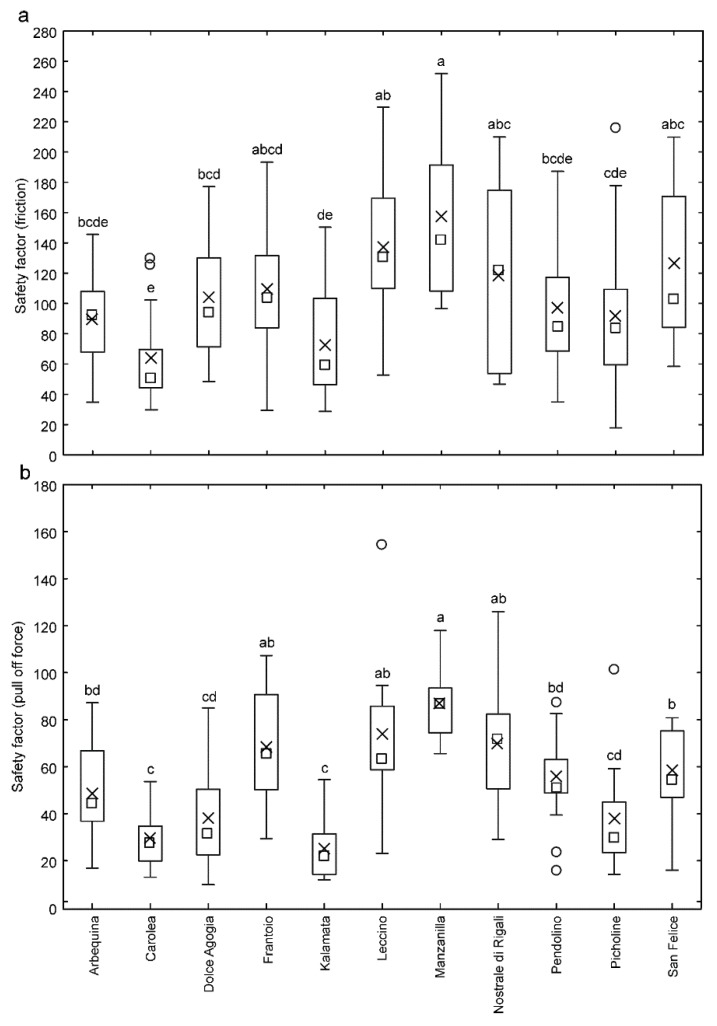
Safety factor (force divided by the insect weight) related to friction force **(a)** and pull off force **(b)** of *Bactrocera oleae* females on the fruit surface of different cultivars of *Olea europaea*. Boxplots show the interquartile range and the median, whiskers indicate 1.5 x interquartile range, “X” shows the arithmetic mean and circles show outliers. Boxplots with different lower-case letters are significantly different at *p* < 0.05, one-way repeated measures analysis of variance (ANOVA), Tukey’s HSD post-hoc test.

The friction experiments with *B. oleae* females on the ripe fruit surface of different cultivars of *O. europaea* showed that the safety factor (force divided by the weight of insect individual) obtained from the friction force varied significantly depending on the olive cultivar (*F* = 7.85, *d.f.* = 14, 10, 140, *p* < 0.0001). Among the tested cultivars, the highest safety factor was recorded on Manzanilla cultivar, together with Nostrale di Rigali, Leccino, Frantoio and San Felice, while the lowest safety factor was recorded on Carolea, together with Picholine, Arbequina, Kalamata and Pendolino. In Dolce Agogia, it was intermediate (Figure 4a). 

The safety factor obtained from the pull off force also varied significantly depending on the olive cultivar (*F* = 18.67, *d.f.* = 14, 10, 140, *p* < 0.0001). Among the tested olive surfaces from different cultivars, the highest safety factor was recorded on the Manzanilla cultivar, together with Nostrale di Rigali, Leccino and Frantoio, while the lowest safety factor was recorded on Carolea, together with Kalamata, Dolce Agogia and Picholine. In Arbequina, Pendolino and San Felice, it was intermediate (Figure 4b). 

The safety factor values obtained on glass at the beginning (friction: 133.4 ± 9.7; pull off: 39.7 ± 2.0) and at the end (friction: 162.3 ± 20.2; pull off: 35.9 ± 2.5) of experiments with each set of olives were not significantly different for both friction (*t* = 1.66, *d.f.* = 14, *p* = 0.1193) and pull off forces (*t* = 1.64, *d.f.* = 14, *p* = 0.1223). 

**Figure 5:**
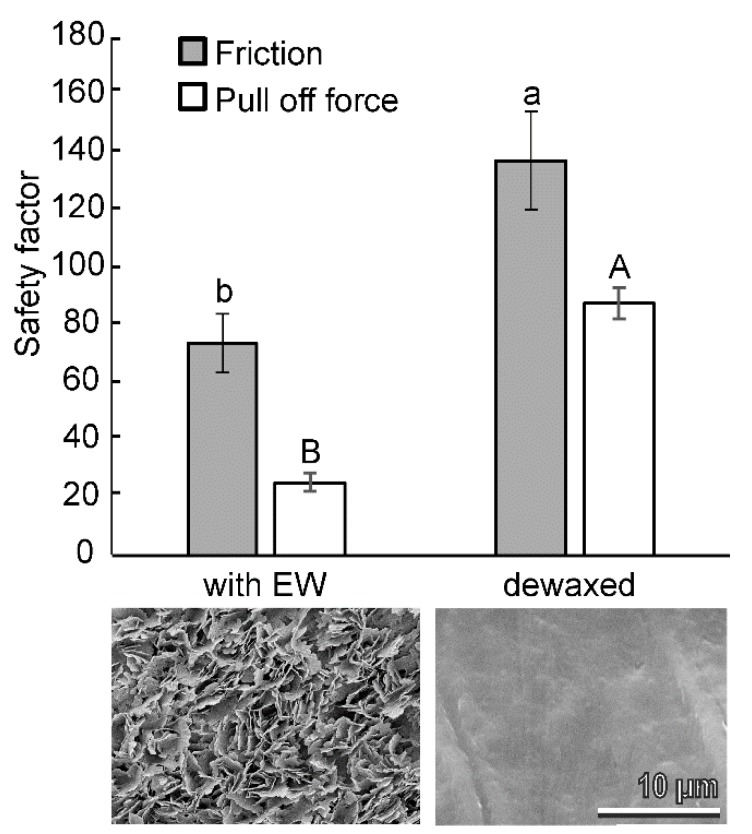
Safety factor (force divided by the insect weight) based on measured friction and pull off forces of *Bactrocera oleae* females on the olive fruit surface of the cultivar Kalamata tested intact (with EW) and with mechanically removed waxes (dewaxed). Bars with different upper-case letters and lower case letters are significantly different at *p* < 0.05, respectively, *t*-test for dependent samples. Images show the corresponding olive fruit surfaces in cryo-SEM.

In the experiments, aiming to compare the attachment ability (friction and pull off forces) of *B. oleae* females on the fruit surface with intact EWs, and with mechanically-removed waxes (dewaxed) (Figure 5), the safety factor was significantly higher on dewaxed olives than on intact olives for both friction (*t* = 3.01, *d.f.* = 12, *p* = 0.0108) and pull off forces (*t* = 10.14, *d.f.* = 14, *p* < 0.0001) (Figure 5). The contact angle of the intact olive surface was significantly higher than that of the dewaxed olive surface of the same cultivar (140.5 ± 3.5° and 107.8 ± 0.8°, respectively; *t* = 10.58, *d.f.* = 18, *p* < 0.0001).

**Figure 6:**
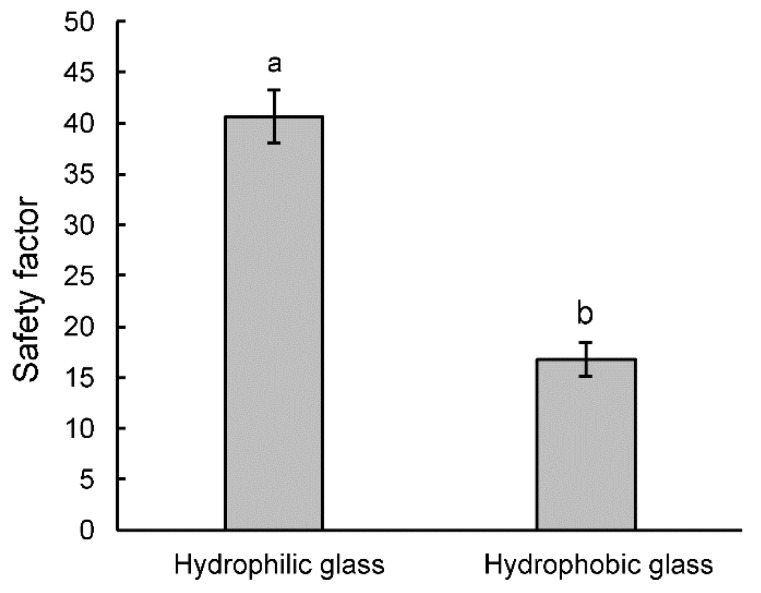
Safety factor (force divided by the insect weight) based on measured traction forces of *Bactrocera oleae* females obtained on hydrophobic and hydrophilic glass. Bars with different letters are significantly different at *p* < 0.05, *t*-test for dependent samples.

In the traction experiments on hydrophilic glass (contact angle: 19.94 ± 0.41°) and hydrophobic glass (contact angle: 112.62 ± 1.50°), the safety factor varied significantly depending on the type of the tested surface (*t* = 11.26, *d.f.* = 9, *p* < 0.0001). It was higher on hydrophilic glass than on hydrophobic glass (Figure 6). 

**Figure 7:**
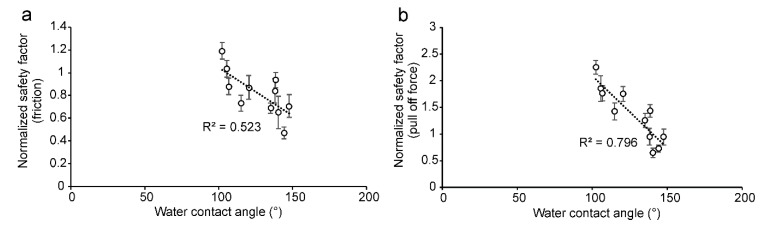
Relationship between the water contact angle on olive fruit surfaces of different cultivars of *Olea europaea* and the normalized safety factors relative to friction force (**a**) and pull off forces (**b**) of *Bactrocera oleae* females.

The normalized safety factor (safety factor normalized on that obtained on glass, to reduce the variability caused by individual insects) related to friction force (Figure 7a) and pull off force (Figure 7b) of *B. oleae* females obtained in our force experiments on the olive fruit surface of the different cultivars of *O. europaea* was negatively correlated with the water contact angle on the olive surfaces (friction force: *F* = 9.87, *d.f.* = 1, 9, *p* = 0.0119; pull off force: *F* = 35.08, *d.f.* = 1, 9; *p* = 0.0002).

## 4. Discussion

### 4.1. EWs Effect on the Attachment Ability of *Bactrocera oleae* to Olive Fruit Surfaces

The importance of 3D EWs in decreasing surface wettability is known [2,8], and their role in reducing insect attachment to the plant surface has been studied in many insect and plant species using different experimental approaches [27,28,29,30,31] (see also reviews in [10,11,12]). In our recent investigation, testing the attachment ability of females of another Tephritidae, the Mediterranean fruit fly *Ceratitis capitata* Wiedemann (Diptera: Tephritidae) to fruits of various host plants characterized by different surface morphology (smooth, hairy, waxy) [32], a strong reduction in attachment ability was recorded on *Prunus domestica*, compared with other tested fruit surfaces, due to the dense and regular 3D EW coverage. This last in *P. domestica* is composed of numerous, very short, thin-walled tubules oriented at different angles to the surface, making the fruit surface superhydrophobic. The comparison between the attachment ability to waxy surfaces of *C. capitata*, which is a typical poliphagous species infesting in more than 300 plant species their fruits and nuts [33,34] having different physico-chemical properties, and the olive fruit fly, which is a monophagous species feeding exclusively on wild and cultivated olives [13] typically characterized by hydrophobic surface, is particularly interesting, in relation to possible adaptations to the trophic niche. Both insect species show morphologically similar attachment organs represented by pretarsal paired pulvilli covered by hundreds of capitate “tenent setae” with different terminal plate shapes in the proximal and distal portions of the pulvillus, which is a typical condition described in other brachyceran families, such as Calliphoridae [35,36,37,38,39,40] and Syrphidae [38,41]. As far as their attachment ability is concerned, the experiments demonstrated that fruit EW plays an important role in reducing attachment, not only in *C. capitata*, but also in *B. oleae*, since the attachment ability of this insect to a dewaxed olive (Kalamata) (contact angle of 107.8 ± 0.8°) was higher than that to the intact olive of the same cultivar (contact angle of 140.5 ± 3.5°). 

In our experiments, the safety factor values of *B. oleae* obtained on glass at the beginning and at the end of each set of tests with olives were not significantly different. Similarly, in force experiments with the beetle *Chrysolina fastuosa* (Scopoli) (Coleoptera: Chrysomelidae) [42], with the aphid *Acyrthosiphon pisum* Harris (Hemiptera: Aphididae) [43] and with the bug *Nezara viridula* L. (Hemiptera: Pentatomidae) [44] on plant species with EWs, no influence of the waxy surfaces on the subsequent insect attachment ability was observed, or recovery of the attachment ability occurred rather quickly. Therefore, we can exclude that one of the main reasons of the reduction in *B. oleae* attachment to the olive surface could be linked to contamination of insect adhesive pads with broken fragments of wax projections, as demonstrated previously for several insect species with the hairy type of pads, such as *Adalia bipunctata* (L.) (Coleoptera: Coccinellidae) [45] or *C. fastuosa* [46]. The reduction of insect adhesion of *B. oleae* could be linked better to the roughness hypothesis [42,47], stating that wax projections reduce insect adhesion owing to the surface microroughness, similar to the effect of microrough polishing paper with 0.3–1.0 μm asperity sizes minimizing insect pad contact areas due to small surface irregularities. Such a reduction has been demonstrated in many different insect species [44,48,49,50,51,52,53,54,55], among which the Mediterranean fruit fly *C. capitata* [32].

Our experiments with smooth, artificial surfaces also confirmed an important role of surface wettability in affecting *B. oleae* attachment. Indeed, a reduction of traction force of *B. oleae* on a hydrophobic surface compared with a hydrophylic one was clearly visible: the safety factor was higher on hydrophilic glass (contact angle = 19.94 ± 0.41°) than on hydrophobic glass (contact angle = 112.62 ± 1.50°). A significant decrease in the attachment force on artificial surfaces with an increasing surface contact angle has been reported also in another Diptera species, *C. capitata* [32], and in different other insect species belonging to Coleoptera, such as *Gastrophysa viridula* (De Geer) (Coleoptera, Chrysomelidae) [22], *Cylas puncticolis* Boheman (Coleoptera, Brentidae) [56], *Cryptolaemus montrouzieri* Mulsant, (Coleoptera, Coccinellidae) [57] and *Coccinella septempunctata* L. (Coleoptera, Coccinellidae) females [24,58] as well as to Heteroptera, such as *N. viridula* [55]. These results are explained by the reduction of the attachment force on microstructured substrates owing to the reduced role of the adhesive fluid in generation of capillary forces, by either too low or too strong affinity of the insect adhesive fluid to the microstructured substrates [59]. However, in the beetle *Galerucella nymphaeae* (L.) (Coleoptera: Chrysomelidae) [60], insects showed the highest forces on rather smooth surfaces with a water contact angle of 83° (similar to that of the host plant), while hydrophilic (contact angle of 6° and 26°) and hydrophobic (contact angle of 109°) surfaces caused a reduction of their attachment ability. This suggested that the contact angle of the host plant might rule the attachment behavior of species on different surfaces in relation with adaptation [60]. In this regard, as above reported, the comparison between the attachment ability of two Tephritidae species, such as the polyphagous *C. capitata* [32] and the monophagous *B. oleae*, is particularly interesting. However, the similar results regarding the attachment ability to smooth hydrophilic and hydrophobic surfaces (the same glass surfaces were tested) obtained in these two species do not confirm the hypothesis (at least in Tephritidae) that the effect of the substrate chemistry on insect attachment could depend on insect species and its level of specialization. Further investigations on the tarsal adhesive organs and the attachment ability to hydrophilic or hydrophobic surfaces of other representative insect species, which are more or less specialized, could further clarify this aspect.

### 4.2. Different Attachment Ability of *Bactrocera oleae* to the Olive Fruits of Different Cultivars of *Olea europaea*

EWs appear in many different morphological forms (see review in [1]), whose structure is strictly depending on chemical composition [1,2]. A high variability of olive EW morphology in different cultivars of *O. europaea* has been reported by Lanza and Di Serio [61]. The EW of ripe olive is mainly composed of triterpenic acids, alkanes, alcohols, aldehydes, alkyl esters, benzyl esters, triacylglycerols, and fatty acids [62,63,64,65,66,67]. A chemical analysis of EWs of olives in different cultivars has revealed the presence of various fractions consisting of chemicals from the above reported compound families, whose proportions depended strongly upon the olive cultivar [21,67]. Our data on the attachment ability of *B. oleae* females to the ripe fruit surface of different cultivars of *O. europaea* revealed that both friction force (force preventing sliding of two contacting bodies) and pull off force (force resisting separation of two contacting bodies) varied significantly depending on the olive cultivar and these effects were similar for both above forces. Moreover, from our data it emerges that (1) the olive surface in different cultivars is characterized by different mean values of water contact angles ranging from 102.30° (Manzanilla) to 147.62° (Picholine) and (2) there is a negative correlation between *B. oleae* attachment ability and the olive surface contact angle mean value. 

The EW coverage of ripe olives in different cultivars of *O. europaea* examined in the present study is represented by complex 3D structures in the form of irregular platelets. These EWs appear much more similar in the different cultivars than those described by Lanza and Di Serio [61], but this could be due to the different analyzed cultivars (only one was common in both investigations). For the only common Kalamata cultivar, our results on the micromorphology of the EW coverage are on the whole in line with the previous study [61] and some differences could be explained by the different stage of olive ripening used (ripe *vs* green) or different fixation method employed (cryo fixation vs 3–5 h in the oven at 30 °C). However, different cultivars in our study showed some variability in the orientation of the EW platelets. Most of them were oriented perpendicularly to the olive surfaces (e.g., in Picholine), but in some cultivars (especially those revealing a higher wettability by water, such as Manzanilla), wax platelets were oriented at rather shallow angles. In the first case, wax projections created more distinct surface microroughness, responsible for higher hydrophobicity of these surfaces. On fruits of these cultivars, the tested insects showed the poorest attachment (the lowest safety factors for both friction and pull off forces). In the second case, more flattened microrough fruit surfaces having lower hydrophobic properties represented more suitable substrates for the attachment of the olive fruit fly (significantly higher safety factors for both friction and pull off forces). The different angle of wax projection orientation might influence not only the wettability of the surface by different fluids, but also the amount of the direct real contact between terminal contact elements of the fly tenent setae and olive surface. This latter effect might be explained by different relative relationships between dimensions of terminal contact elements of fly tenent setae and substrate asperities. Vertically oriented wax projections generate very small asperities of high amplitude, whereas flattened wax projections produce larger asperities with relatively low amplitude. As previously demonstrated in different theoretical considerations, the first type of substrate will much stronger reduce real contact area with the terminal contact elements of the fly setae and in turn much stronger reduce fly adhesion than the second type of substrate [49,51,54].

Considering that insect attachment depends not only on the presence of 3D wax, but also on the projection size, density of the EW coverage or distribution of individual projections, as shown for the ladybird beetle *C. montrouzieri* walking on *Pisum sativum* plants with wild-type waxes and with reduced waxes caused by mutation [68] and for the ladybird beetle *C. septempunctata* on bio-inspired wax surfaces formed by four alkanes of varying chain length [69], we suggest that a different pattern of the fruit EWs in the different cultivars of *O. europaea* creates a different microroughness and produces a different level of wettability and different amount of direct real contact area between terminal contact elements of the fly setae and EW projections, thus effecting attachment ability of *B. oleae* females to the olive surface, as demonstrated in the present study. 

### 4.3. Attachment Ability of *Bactrocera oleae* and Cultivar Susceptibility to the Olive Fruit Fly

The factors underlining the tolerance of the different cultivars of *O. europaea* to the olive fruit fly are complex [70] and may be due to different factors, such as mechanical barriers, chemical or morphological features and their combination. The role of EWs in reducing *B. oleae* oviposition has been demonstrated in previous investigations [18,19], even if the susceptibility to the fly of the different cultivars in relation with the olive EW coverage still needs to be clarified. According to Olive Germplasm database [71], an increasing level of susceptibility to *B. oleae* can be attributed to the cultivars tested in the present study, in the following order (from the lower to the higher): Kalamata, Picholine, Pendolino, Nostrale di Rigali, Leccino, San Felice, Frantoio and Manzanilla. This trend fits well to the different levels of *B. oleae* attachment ability to the olive fruit recorded in the present investigation. Such a correspondence is less clear for Arbequina, Carolea and Dolce Agogia (high susceptibility and low *B. oleae* attachment ability), probably owing to other factors acting together with the attachment ability of the fly and affecting the resistance properties of different cultivars. Further studies are necessary to better clarify these aspects.

## 5. Conclusions

The research efforts aimed to investigate the interaction between the olive and its key enemy are relatively few compared with the economic impact of *B. oleae* [17]. In this context, studies clarifying the attachment ability of *B. oleae* in relation to the different physico-chemical features of the EW in different *O. europaea* cultivars could contribute to deepen the knowledge about this important insect pest, thus helping to develop control methods alternative to the use of pesticide harmful for human health. In particular, starting from the results of the present investigation highlighting that *B. oleae* friction force and pull off force on the olive fruit surface varied significantly depending on the olive cultivar, in consideration that EW morphology and chemical composition can change during fruit development [21,62,72,73] and could be affected by environmental abiotic factors potentially changing the surface micro- and nanostructure that in turn influence the degree of porosity, wettability of surface as well as the ability of the fly terminal contact elements to form real contact area with these surfaces [10], further investigations on the mechanical ecology of the olive fruit fly adhesion to the olive fruit surface are advisable.

## Figures and Tables

**Table 1:** Area of single pulvillus in the different legs of female and male *Bactrocera oleae*.

Legs	Pulvillus Area (µm^2^)	n
♀ *		
foreleg	10440 ± 234.0 ^b^	10
midleg	12644 ± 353.4 ^a^	10
hindleg	14118 ± 340.5 ^a^	10
♂		
foreleg	7437 ± 236.9 ^A^	10
midleg	8879 ± 292.8 ^B^	10
hindleg	9512 ± 284.5 ^B^	10

Data are presented as mean ± the standard error of the mean (s.e.m.). The asterisk and different letters show statistical differences at *p* < 0.05, two-way repeated measures ANOVA.

**Table 2:** Contact angles of water on olive fruit surface of different cultivars.

Cultivars	Contact Angle (°)	n
Arbequina	135.18 ± 5.79 ^abc^	10
Carolea	144.18 ± 3.07 ^a^	11
Dolce Agogia	138.16 ± 5.59 ^ab^	9
Frantoio	120.40 ± 4.41 ^bd^	10
Kalamata	140.48 ± 3.50 ^ab^	10
Leccino	105.62 ± 5.95 ^d^	11
Manzanilla	102.30 ± 3.72 ^d^	10
Nostrale	106.88 ± 3.67 ^d^	8
Pendolino	115.02 ± 5.05 ^cd^	8
Picholine	147.62 ± 4.72 ^a^	10
San Felice	138.59 ± 3.92 ^ab^	9

Data are presented as mean ± s.e.m. Different letters show statistical differences at *p* < 0.05, one-way ANOVA.
